# Differential inflammasome expression and IL-1β secretion in monocyte-derived dendritic cells differentiated with IL-4 or IFN-α

**DOI:** 10.1186/1742-6405-10-35

**Published:** 2013-12-27

**Authors:** Alessandra Pontillo, Bruna T Santillo, Alberto JS Duarte, Telma M Oshiro

**Affiliations:** 1 Laboratory of Immunogenetics, Departament of Immunology, Institute of Biomedical Sciences, University of Sao Paulo, Sao Paulo, Brazil; 2 Laboratory of Medical Investigation in Dermatology and Immunodeficiency, LIM-56, Faculty of Medicine, University of Sao Paulo, Sao Paulo, Brazil

**Keywords:** HIV-1, Type-1 IFN, Dendritic cells, Inflammasome, NALP3, IL-1β, Vaccine, Immunotherapy

## Abstract

**Background:**

NLRP3-inflammasome activation was evaluated in monocyte-derived dendritic cells (DC) obtained through IL-4 (IL4-DC) or IFN-α (IFN-DC) protocols and pulsed with chemically inactivated HIV-1. Inflammasome’ genes expression and IL-1β secretion were compared in DC isolated from 15 healthy subjects (HC) and 10 HIV-1 infected individuals (HIV+).

**Findings:**

Whether HIV was able to increased NLRP3-inflammasome genes expression and IL-1β secretion in IL4-DC from HC, the induction of inflammasome appeared significantly reduced in IFN-DC from HC, suggesting a different responsive state of IFN-DC compared to IL4-DC. No inflammasome activation was observed in IL4-DC as well as in IFN-DC derived from HIV + subjects, confirming previous findings on “unresponsive” state of DC derived from HIV + possibly due to chronic inflammatory state of these individuals.

**Conclusions:**

Our results showed that IFN-α differently modulates inflammasome expression during monocytes-DC in vitro differentiation. These findings could be of interest considering the on-going research about DC manipulation and therapeutic strategies for HIV + involving DC-based immune-vaccines.

## Findings

### Background and aim

In the past ten years an increasing number of dendritic cell (DC)-based therapeutic vaccination trials against HIV-1 has been developed augmenting the general interest in dendritic cell biology and in the interaction between this innate immune cell and HIV-1 [1].

There are at least two major subtypes of DC: myeloid and plasmacytoid DC. Myeloid DC (mDC) are professional antigen presenting cell capable to initiate cellular immune response. On the other hand plasmacytoid DC (pDC) exhibit an exuberant anti-viral response through the production of high levels of IFN-alpha [2].

In HIV infection it has been demonstrated a reduction in absolute number of mDC and pDC, especially in patients with active HIV-1 replication [3], suggesting a key role of DC in controlling viral load. Moreover the preserved functionality of pDC isolated from “elite controllers” may be one of the mechanisms involved in the control of HIV-1 viremia in these subjects [4].

Myeloid DC obtained culturing peripheral blood monocytes with IL-4 and granulocyte-macrophage colony-stimulating factor (GM-CSF) (IL4-DC) have been implied in immunotherapy of cancer and, most recently, of HIV infection [5].

Alternative protocols for maturation of DC included the use of interferon (IFN)-α instead of IL-4 (IFN-DC). Type I interferons markedly promotes the differentiation of peripheral blood monocytes into DC. IFN-DC are able to induce a strong cytotoxic response and cross priming of CD8+ T cells against viral or tumoral antigens both *in vitro* and *in vivo*[6,7]. IFN-DC exhibit a combined phenotype of mDC and pDC associated with characteristics of natural killer (NK) cells [8,9].

In the context of therapeutic vaccine against HIV-1, the possibility of using IFN-DC would provide several advantages, including augmented antigen presentation, cytotoxic function and type I IFN production.

Considering the key role of NLRP3-inflammasome during maturation and activation of DC in mice [10], our group has recently started working on inflammasome activation in DC obtained *in vitro* from peripheral blood monocytes. DC response to HIV-1 through NLRP3-inflammasome could be important in HIV vaccine development especially in DC-based immune treatment.

We demonstrated that human monocyte-derived IL4-DC presented an inducible activation of NLRP3-inflammasome by lipopolysaccharide/LPS as well as by HIV-1 [10]. It is interesting to note that the same differentiation protocol used in monocytes isolated from HIV + individuals (HIV-DC) showed a different response. NLRP3-inflammasome was not induced by LPS or HIV in HIV-DC, however its basal activation resulted higher in these cells compared with DC from healthy subjects (HC-DC), letting us hypothesize an “unresponsive” condition of HIV-DC that may affect vaccine preparation [10].

In this study we investigate the rate of inflammasome expression and activation in monocyte-derived IFN-DC obtained from healthy individuals and HIV + patients. Our findings could be of interest considering the on-going research about DC manipulation and therapeutic strategies for AIDS involving DC-based immune-vaccines.

### Individuals and methods

15 healthy subjects (HC, 7 males, 8 females; mean age 30 ± 5 standard deviation/SD) and 10 HIV-1-positive volunteers (HIV+, 8 males, 2 females; mean age 28 ± 2 SD) were recruited from “Hospital das Clinicas” (University of São Paulo, Brazil). HIV + patients had a CD4+ lymphocytes count > 500 cells/μl and were “naïve” for treatment. All the patients have been infected for more than 5 years and they do not presented specific clinical symptoms at the moment of recruitment. Written informed consent was obtained according to the protocol of “Hospital das Clinicas” Ethical Committee (CAPPesq) (São Paulo, Brazil).

Monocytes were isolated by adherence from peripheral blood monocytes obtained by centrifugation over Ficoll-Paque gradient and cultured at 1.5-2 × 10^6^/ml in AIM-V medium (*Gibco*) containing 50 ng/ml GM-CSF (*Peprotech*) and 50 ng/ml IL-4 (*Peprotech*) or IFN-α (Schering-Plough) (IL4-DC and IFN-DC, respectively). On day 5, DC were pulsed with 0.5 × 10^9^ particles of alditrithiol-2 inactivated HIV (as reported in [10]) for 4 hours. Supernatants were harvested and used for IL-1β secretion dosage; cells were lysed for mRNA isolation and gene expression analysis.

Total RNA was isolated using the RNAqueous micro kit (*Ambion, Life Technologies*) and retro-transcribed with the SuperScritp-II kit (*Invitrogen*). *NLRP1, NLRP3, NLRC4, AIM2, CASP1, IL1B* genes were amplified with specific TaqMan® Gene Expression Assays (*Applied Biosystems*) using the ABI 7300 platform (*Applied Biosystems*). *ACTB* was the housekeeping gene used for normalization. Fold-change (FC) genes expression was calculated comparing stimulated (+HIV) and untreated/resting cells as 2^-∆∆Ct^ following the indications of Livak et al. [11].

The secreted IL-1β was evaluated with ELISA (*BD Biosciences Pharmigen)*. Results were expressed as pg/ml.

One-way Anova analysis was used to compare different groups and a significance level of 0.05 was applied. Prism 5.01 software (GraphPad Software, Inc) was used for done graphs and statistical analysis.

## Results and discussion

We evaluated the expression of *NLRP1*, *NLRP3, NLRC4, AIM2, CASP1, IL1B* genes in DC from 15 healthy subjects obtained through IL-4 or IFN-α differentiation protocol and pulsed with chemically inactivated HIV-1. IL-1β secretion was also evaluated as marker of inflammasome activation.

Cells differentiation state was checked by flow cytometry looking at granulosity and common DC markers (Additional file 1: Figure S1). No significant differences were observed in differentiation markers between IL4-DC and IFN-DC.

When genes expression was evaluated the following criteria were applied: only samples with Ct < 35 and with a positivity of at least 80% of the samples were included in the analysis; normalized data (∆Ct) must be homogenous for all the genes in each group (t test p-value > 0.05); FC > 1.5 or <0.5 were taken in account as up-regulation or down-regulation.

**Figure 1:**
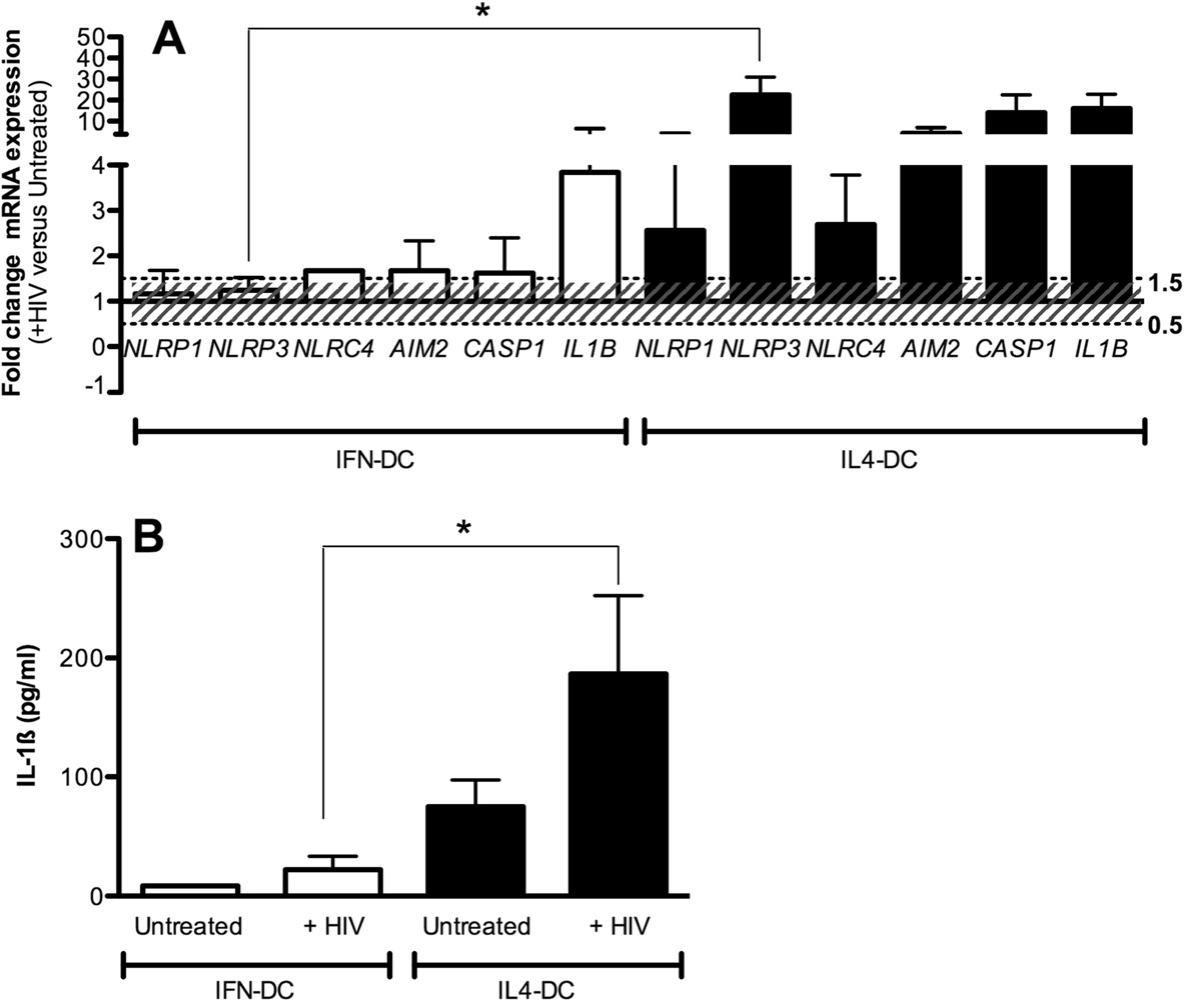
**Inflammasome genes expression in IL4-DC and IFN-DC from healthy subjects pulsed with HIV-1.***mRNA* expression 4 hours after stimulation of IL4-DC and IFN-DC with chemically inactivated HIV-1 was reported for *NLRP1, NLRP3, NLRC4, AIM2, CASP1* and *IL1B* genes **(1A)***.* Fold-change (FC) in mRNA expression of each gene was calculated with respect to the untreated condition as 2^-∆∆Ct^ where ∆∆Ct = ∆Ct HIV- ∆Ct Untreated. Results are expressed as average (AVG) of 2^-∆∆Ct^ ± standard error, n = 20. All the Ct values were normalized against *ACTB* Ct (∆Ct). Stacked lines showed FC threshold (0.5-1.5). 1-way Anova analysis was used to compare genes expression in IFN-DC and IL4-DC. * = p < 0.05. **(1B)** Supernatants of IFN-DC and IL4-DC stimulated or not with HIV-1 were analysed for the presence of secreted IL-1β. Results are expressed as the average concentration (pg/ml) ± standard error. 1-way Anova analysis was used to compare IFN-DC and IL4-DC. * = p < 0.05.

HIV induced increased expression (FC > 1.5) of *NLRP1, NLRP3, AIM2, CASP1* and *IL1B* in IL4-DC, and of *NLRC4, AIM2, CASP1* and *IL1B* in IFN-DC (Figure 1A). However the rate of induction appeared to be significantly reduced in IFN-DC compared to IL-4 DC (p < 0.003).

Of note HIV-1 was able to increase *NLRP3* expression in IL4-DC (FC = 22.47), but it was not able to do the same in IFN-DC (FC = 1.24) (p < 0.003) (Figure 1A).

These differences in inflammasome genes expression between IL4-DC and IFN-DC were emphasized by the results obtained from IL-1β secretion analysis in DC supernatants. HIV induced a significant increase of the pro-inflammatory cytokine in IL4-DC (+HIV = 186.5 pg/ml versus Untreated = 74.87 pg/ml; p = 0.026) but not in IFN-DC (+HIV = 21,88 pg/ml versus Untreated = 8,65 pg/ml; p = 0.301) (Figure 1B). Basal secretion of IL4-DC appeared augmented compared to IFN-DC however not in a statistically significant way.

These findings suggested that chemically inactivated HIV-1 is able to induce NLRP3-inflammasome activation in IL4-DC but not in IFN-DC.

As we previously hypothesized [10] NLRP3-inflammasome could play a role in response against HIV and could participate to full dendritic cell activation and to increased proliferation of specific HIV-induced T cells. However our new findings point out that the protocol implied to differentiate monocytes to DC could affect inflammasome biology. Our results agreed with previously reported data by Guarda et al. [12] about the ability of type I IFNs to suppress pro-IL-1 levels and decrease the activity of NLRP1 and NLRP3-inflammasomes in mice. Moreover it is well known that the activation of NLRP3-inflammasome in DC is related with the ability to induce a Th17 polarization in naïve T cells [13], while IFN-DC are able to induce Th1 but fail to polarize naïve T cells into Th17 [14], letting us hypothesize that in IFN-DC the inflammasomes are not induced.

**Figure 2:**
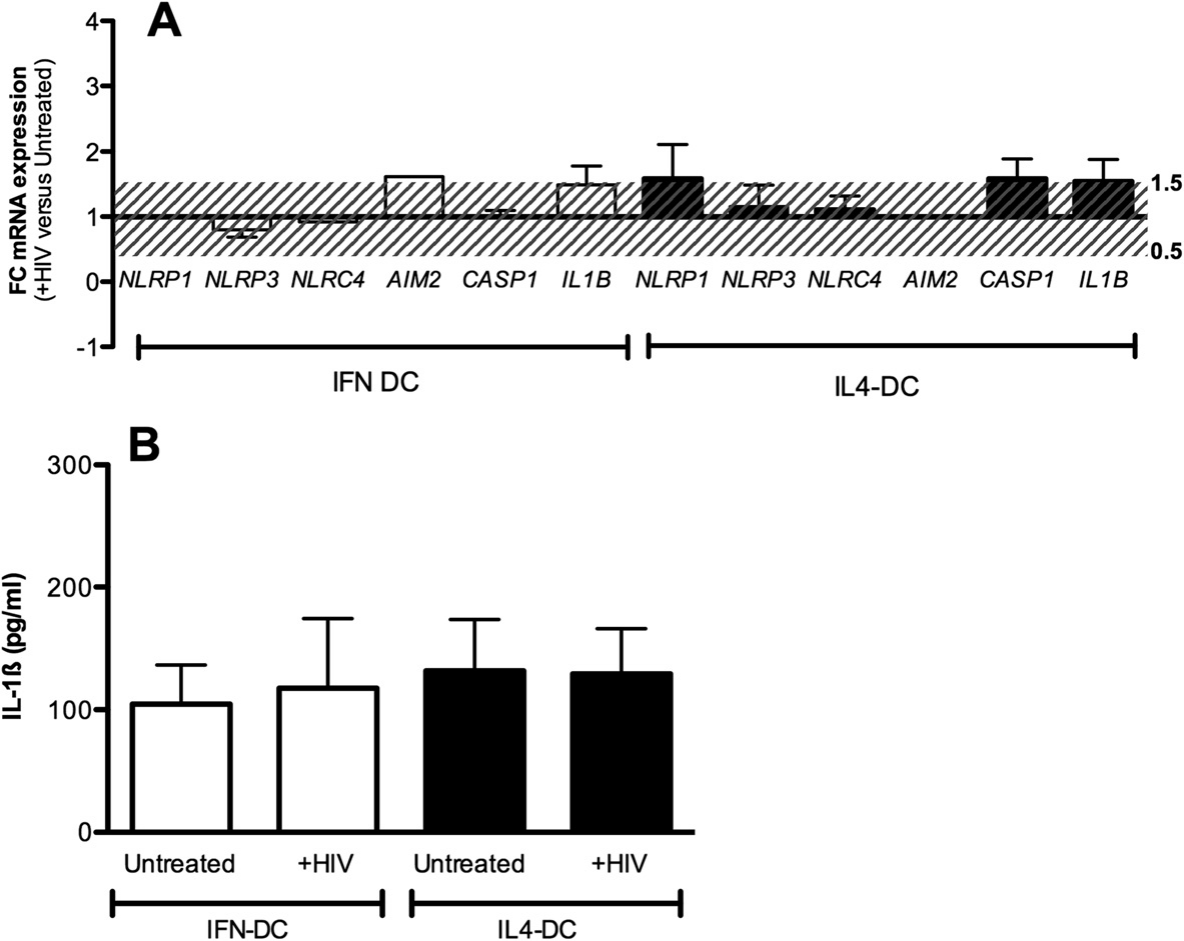
**Inflammasome genes expression in IL4-DC and IFN-DC from HIV + subjects pulsed with HIV-1.***mRNA* expression 4 hours after stimulation of IL4-DC and IFN-DC with chemically inactivated HIV-1 was reported for *NLRP1, NLRP3, NLRC4, AIM2, CASP1* and *IL1B* genes **(2A)***.* Fold-change (FC) in mRNA expression of each gene was calculated with respect to the untreated condition as 2^-∆∆Ct^ where ∆∆Ct = ∆Ct HIV- ∆Ct Untreated. Results are expressed as average (AVG) of 2^-∆∆Ct^ ± standard error, n = 20. All the Ct values were normalized against *ACTB* Ct (∆Ct). Stacked lines showed FC threshold (0.5-1.5). 1-way Anova analysis was used to compare genes expression in IFN-DC and IL4-DC. * = p < 0.05. **(2B)** Supernatants of IFN-DC and IL4-DC stimulated or not with HIV-1 were analysed for the presence of secreted IL-1β. Results are expressed as the average concentration (pg/ml) ± standard error. 1-way Anova analysis was used to compare IFN-DC and IL4-DC.

When DC derived from HIV + individuals were analysed, we observed that HIV was not able to significantly induce any of the inflammasome genes in IL4-DC as well as in IFN-DC (1.5 < FC < 0.5) (Figure 2A). According to our previous published data [10], in untreated cells IL1-β secretion was higher in HIV-DC compared to HC-DC whatever protocol was used (Figure 1B and 2B; p = 0.02). Moreover HIV was not able to induce augmented amount of cytokine in IFN-DC or in IL4-DC (Figure 2B). We have previously hypothesized that IL4-DC from HIV + individuals presented an increased basal activation of NLRP3-inflammasome possibly due to the well-known chronic inflammatory state of HIV + patients leading to an “unresponsive” state of monocyte-derived DC [10]. Our findings emphasized once more that this condition is independent of the protocol used for monocyte-DC differentiation.

We are aware that this study is preliminary and that deep investigation about the functionality of IFN-DC is needed, such as ability to activate a lymphocytes specific response, but we would emphasize the importance of DC differentiation protocol in experimental design. Moreover, considering the on-going trials of DC-based immune treatment against HIV-1 [15] it is interesting to remember that the in vitro manipulation of DC could be a limited step in vaccine preparation. Whether it will be fully demonstrated the role of NLRP3-inflammasome in human DC maturation/activation and the impact of inflammasome induction in lymphocyte specific response, our findings suggest that protocols using IL-4 may represent a better choice than IFN ones.

## Abbreviations

DC: Monocyte-derived dendritic cells (DC); IL: Interleukin; IL4-DC: Monocyte-derived dendritic cells obtained by IL-4 and GM-CSF protocol; IFN-DC: Monocyte-derived dendritic cells obtained by IFN-α and GM-CSF protocol; HC: Healthy subjects; HIV+: HIV-1 infected individuals.

## Competing interests

The authors declare that they have no competing interests.

## Authors’ contributions

A.P. conceived of the study, coordinated its design, performed statistical analysis and wrote the manuscript; B.T.S. carried out the experiments; A.J.S.D. is the responsable of the laboratory and revised the work critically; T.M.H. is the responsable of the work group, contributed in conceiving and designing the study, analysis and interpretation of data and drafting the manuscript. All authors read and approved the final manuscript.

## Authors’ information

A.P., PhD, works on inflammasome genetics and biology; B.T.S. is a pre-PhD student working on DC biology; A.J.S.D., Prof, is the principal investigator of the on-going clinical trial of DC-based immune treatment against HIV-1; T.M.H., PhD, works on DC biology and she’s the supervisor of the group involved in the preparation of DC for anti-HIV immune therapy.

## Supplementary Material

Additional file 1: Figure S1 Characterization of monocyte-derived dendritic cells. Generated immature monocyte-derived dendritic cells obtained through IFN (IFN-DC) (A) or IL-4 (IL4-DC) (B) protocols for common markers expression (CD11c, HLA-DR, CD80, CD86, CD83, CD1a, CCR7). Cells were CD14 negative. Average percentage of positive cells and standard error were reported for healthy individuals (n = 15) stimulated or not with HIV (+HIV and Untreated, respectively). Click here for file

## References

[B1] McMichael AJ Borrow P Tomaras GD Goonetilleke N Haynes BF The immune response during acute HIV-1 infection: clues for vaccine development Nature Reviews Immunol 2010 10 11 23 10.1038/nri2674 PMC3119211 20010788

[B2] Siegal FP Kadowaki N Shodell M Fitzgerald-Bocarsly PA Shah K Ho S Antonenko S Liu YJ The nature of the principal type 1 interferon-producing cells in human blood Science 1999 10 5421 1835 1837 10.1126/science.284.5421.1835 10364556

[B3] Barron MA Blyveis N Palmer BE MaWhinney S Wilson CC Influence of plasma viremia on defects in number and immunophenotype of blood dendritic cell subsets in human immunodeficiency virus 1-infected individuals J Infect Dis 2003 10 1 26 37 10.1086/345957 12508143

[B4] Machmach K Leal M Gras C Viciana P Genebat M Franco E Boufassa F Lambotte O Herbeuval JP Ruiz-Mateos E Plasmacytoid dendritic cells reduce HIV production in elite controllers J Virol 2012 10 8 4245 4252 10.1128/JVI.07114-11 22318133 PMC3318617

[B5] Rinaldo CR Dendritic cell-based human immunodeficiency virus vaccine J Intern Med 2009 10 1 138 158 10.1111/j.1365-2796.2008.02047.x 19093966 PMC2875880

[B6] Lapenta C Santini SM Spada M Donati S Urbani F Accapezzato D Franceschini D Andreotti M Barnaba V Belardelli F IFN-alpha-conditioned dendritic cells are highly efficient in inducing cross-priming CD8(+) T cells against exogenous viral antigens Eur J Immunol 2006 10 8 2046 2060 10.1002/eji.200535579 16856207

[B7] Parlato S Romagnoli G Spadaro F Canini I Sirabella P Borghi P Ramoni C Filesi I Biocca S Gabriele L Belardelli F LOX-1 as a natural IFN-alpha-mediated signal for apoptotic cell uptake and antigen presentation in dendritic cells Blood 2010 10 8 1554 1563 10.1182/blood-2009-07-234468 20009034

[B8] Korthals M Safaian N Kronenwett R Maihöfer D Schott M Papewalis C Diaz Blanco E Winter M Czibere A Haas R Kobbe G Fenk R Monocyte derived dendritic cells generated by IFN-alpha acquire mature dendritic and natural killer cell properties as shown by gene expression analysis J Transl Med 2007 10 46 10.1186/1479-5876-5-46 17894866 PMC2064912

[B9] Farkas A Kemény L Interferon-α in the generation of monocyte-derived dendritic cells: recent advances and implications for dermatology Br J Dermatol 2011 10 2 247 254 10.1111/j.1365-2133.2011.10301.x 21410666

[B10] Schroder K Tschopp J The inflammasomes [review] Cell 2010 10 821 832 10.1016/j.cell.2010.01.040 20303873

[B11] Pontillo A Silva LT Oshiro TM Finazzo C Crovella S Duarte AJ HIV-1 induces NALP3-inflammasome expression and interleukin-1β secretion in dendritic cells from healthy individuals but not from HIV-positive patients AIDS 2012 10 1 11 18 10.1097/QAD.0b013e32834d697f 21971358

[B12] Livak KJ Schmittgen TD Analysis of relative gene expression data using real-time quantitative PCR and the 2–∆∆CT method Methods 2001 10 402 408 10.1006/meth.2001.1262 11846609

[B13] Guarda G Braun M Staehli F Tardivel A Mattmann C Förster I Farlik M Decker T Du Pasquier RA Romero P Tschopp J Type I interferon inhibits interleukin-1 production and inflammasome activation Immunity 2011 10 2 213 223 10.1016/j.immuni.2011.02.006 21349431

[B14] Mills KH Dungan LS Jones SA Harris J The role of inflammasome-derived IL-1 in driving IL-17 responses J Leukoc Biol 2013 10 4 489 497 10.1189/jlb.1012543 23271701

[B15] García F Routy JP Challenges in dendritic cells-based therapeutic vaccination in HIV-1 infection Workshop in dendritic cell-based vaccine clinical trials in HIV-1 Vaccine 2011 10 38 6454 6463 10.1016/j.vaccine.2011.07.043 21791232

