# Supplementary figures and images for: Differential inflammasome expression and IL-1β secretion in monocyte-derived dendritic cells differentiated with IL-4 or IFN-α
Source: AIDS Res Ther. 2013 Dec 27;10:35. doi: 10.1186/1742-6405-10-35 (PMC3892092; doi:10.1186/1742-6405-10-35)

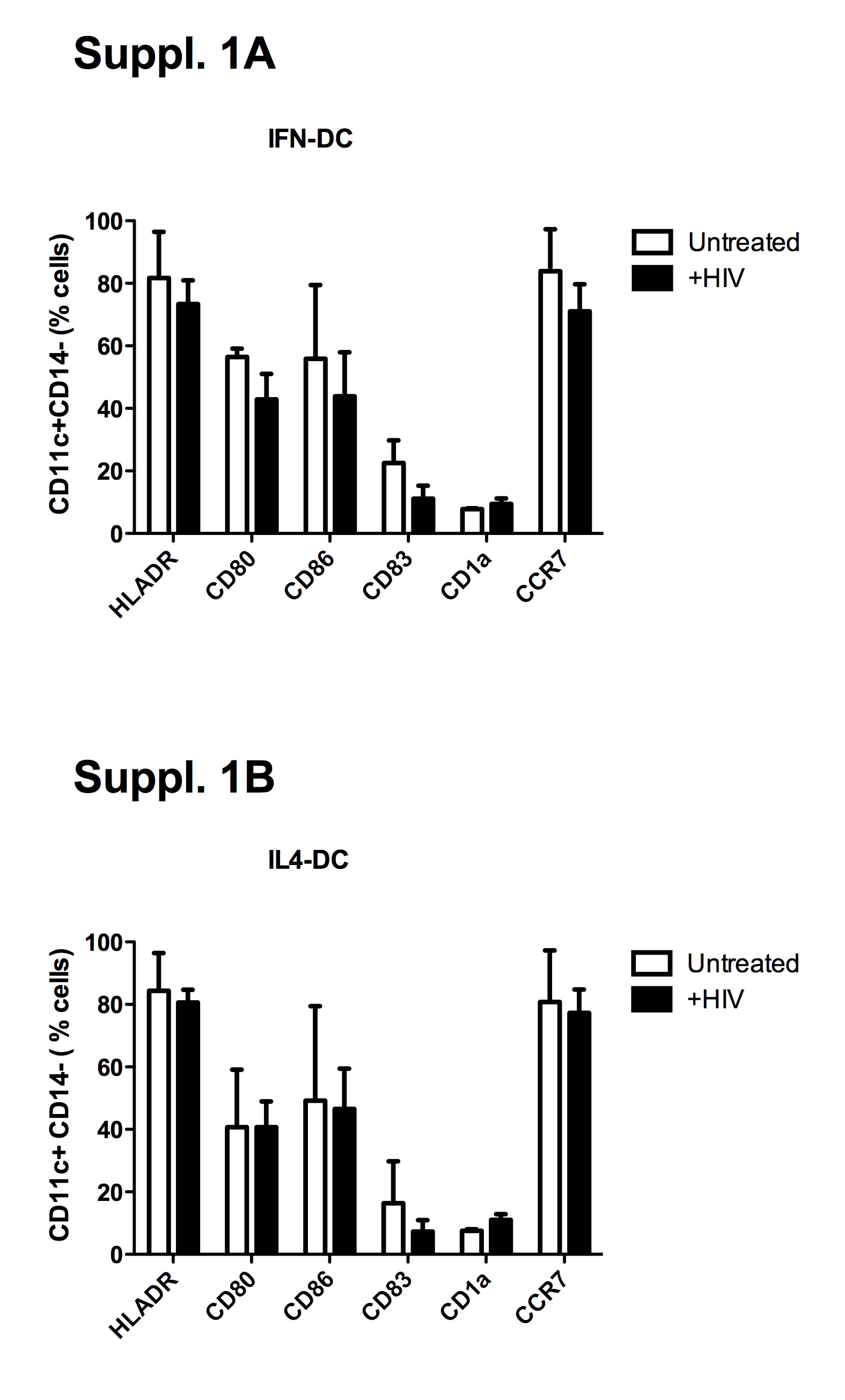

Supplement: Additional file 1: Figure S1 — Characterization of monocyte-derived dendritic cells. Generated immature monocyte-derived dendritic cells obtained through IFN (IFN-DC) (A) or IL-4 (IL4-DC) (B) protocols for common markers expression (CD11c, HLA-DR, CD80, CD86, CD83, CD1a, CCR7). Cells were CD14 negative. Average percentage of positive cells and standard error were reported for healthy individuals (n = 15) stimulated or not with HIV (+HIV and Untreated, respectively). [file 1742-6405-10-35-S1.jpeg]
